# Myasthenia gravis complement activity is independent of autoantibody titer and disease severity

**DOI:** 10.1371/journal.pone.0264489

**Published:** 2022-03-15

**Authors:** Miriam L. Fichtner, Michelle D. Hoarty, Douangsone D. Vadysirisack, Bailey Munro-Sheldon, Richard J. Nowak, Kevin C. O’Connor

**Affiliations:** 1 Department of Neurology, Yale School of Medicine, New Haven, Connecticut, United States of America; 2 Department of Immunobiology, Yale School of Medicine, New Haven, Connecticut, United States of America; 3 UCB Pharma, Cambridge, Massachusetts, United States of America; Istanbul Universitesi Istanbul Medical Faculty, TURKEY

## Abstract

Acetylcholine receptor (AChR) autoantibodies, found in patients with autoimmune myasthenia gravis (MG), can directly contribute to disease pathology through activation of the classical complement pathway. Activation of the complement pathway in autoimmune diseases can lead to a secondary complement deficiency resulting in reduced complement activity, due to consumption, during episodes of disease activity. It is not clear whether complement activity in MG patients associates with measurements of disease activity or the titer of circulating pathogenic AChR autoantibodies. To explore such associations, as a means to identify a candidate biomarker, we measured complement activity in AChR MG samples (N = 51) using a CH50 hemolysis assay, then tested associations between these values and both clinical status and AChR autoantibody titer. The majority of the study subjects (88.2%) had complement activity within the range defined by healthy controls, while six patients (11.8%) showed reduced activity. No significant association between complement activity and disease status or AChR autoantibody titer was observed.

## Introduction

The most common subtype of autoimmune myasthenia gravis (MG) is characterized by pathogenic autoantibodies targeting the nicotinic acetylcholine receptor (AChR) at the neuromuscular junction [1]. These autoantibodies directly contribute to disease pathology primarily, though not exclusively, through activation of complement. The complement system is part of the innate immune system and an important link between the innate and adaptive immune response [2, 3]. Three different activation pathways and over thirty different proteins are associated with the complement system [4]. The three different complement activation pathways, namely the classical, alternative and lectin pathway, differ in their initial steps, but all converge at the C3 activation step [5]. The classical pathway is activated when C1q binds to antibody–antigen complexes [6], the alternative pathway is the result of spontaneous hydrolysis of C3 which can lead to rapid complement activation on foreign cell surfaces [7] and the lectin pathway is activated by the mannose binding lectin (MBL) complex recognizing carbohydrates like mannose on the cell surface of pathogens [8].

Deficiencies of the complement system can be categorized as either hereditary or secondary [9, 10]. The prevalence of both deficiencies can be increased in autoimmune disorders in comparison to the general population [10–12]. Secondary deficiencies are the result of increased complement consumption and subsequent reduced complement activity in either an acute process like septic shock [13] or in autoantibody-mediated autoimmune diseases that involve immune-complexes [10]. Several autoimmune diseases including rheumatoid arthritis [14, 15], ANCA-associated vasculitis [16, 17], systemic lupus erythematosus (SLE) [18, 19] and MG [20, 21] can exhibit reduced complement activity due to its increased consumption during episodes of disease activity. In AChR MG patients the reduced activity can associate with localized autoantibody-mediated complement activation at the neuromuscular junction [22–26].

AChR autoantibodies are useful as a diagnostic biomarker. However, their titer, at single time points, does not correlate well with the disease severity and consequently response to treatment [27–30]. Thus, there is a need for reliable biomarkers in MG to follow the disease course, better inform therapeutic decisions and follow response to therapy. It is not clear whether complement activity in MG patients associates with measurements of disease activity or the circulating levels of pathogenic autoantibodies. To explore a candidate MG biomarker, we measured complement activity and investigated associations with disease burden and AChR autoantibody titers in AChR MG patients and controls.

## Materials and methods

### Patients, controls, and sample handling

This study was approved by the Human Investigation Committee at the Yale School of Medicine (clinicaltrials.gov || NCT03792659). Informed written consent was obtained from all patients. Peripheral blood was collected from AChR MG patients and healthy controls (HC). All AChR MG patients met definitive diagnostic criteria for MG, including positive serology for AChR autoantibodies. We included 40 different unique patients and longitudinally collected samples from some patients resulting in a total of 51 AChR MG samples (mean age: 59.3 +/- 18.8 yrs) and 20 unique HC samples (34.4 +/- 13.3 yrs) (Table 1). The treatment status of the MG patient cohort (N = 51) was heterogeneous: immunotherapy naïve without any prior treatment (patients that never received treatment for MG or any other autoimmune disease; N = 22), no current therapy, but with prior treatment (specifically, patients that were, at the time of collection and three months prior, not receiving any current treatment for MG; N = 4), cholinesterase inhibitor (N = 10), corticosteroids (N = 10), both corticosteroids and azathioprine (N = 1), both corticosteroids and cholinesterase inhibitor (N = 2), IVIg (N = 1), or PLEx (N = 1). Several studies indicate an effect of IVIg on complement activity [31, 32], but this is effect reverts to normal values around 2 weeks after treatment [32]. Therefore, serum samples were only included if the patient did not receive IVIg within the last two weeks. Patient and healthy control sera were processed within 1 h after collection. Clotting was allowed to proceed for 30 min in the collection tune (BD Vacutainer^®^, Serum Blood Collection tube, catalog # 366431), then the tube was centrifuged at 2000 g for 15 min at 4°C. Serum was aliquoted and stored at -80°C before use. The sera were thawed and kept on ice on the day of the experiment. The burden of disease at time of collection was measured by MG Composite (MGC) score. The MGC score is used to measure the clinical status of MG patients by assessing the extent of muscle weakness [33].

**Table 1:** Clinical characteristics and demographics of MG patients.

Characteristics	Number
Samples	51
Patients	40
Male / female	26 / 14
Age, yrs (with std. dev.)	59 (+/- 18.8)
Thymectomy	17
Thymoma	4
Thymus hyperplasia	7
Early Onset MG	17
Late Onset MG	34
MGFA classification	
I	21
II	12
III	7
IV	1
V	2
No current symptoms	8
Treated	25
Prednisone	10
Pyridostigmine	10
Prednisone + Azathioprine	1
Prednisone + Pyridostigmine	2
PLEx	1
IVIg	1
Untreated	26
Immunotherapy naïve	22
No current therapy	4

Abbreviations: yrs, years; MGFA, Myasthenia Gravis Foundation of America; PLEx, plasma exchange; IVIg, intravenous immunoglobulin. No. or mean +/- SD (range) are shown.

### CH50 hemolysis assay

The CH50 hemolysis assay measures the complement activity of the antibody-dependent classical complement pathway by measuring the capability of the serum complement to lyse antibody-sensitized sheep red blood cells (RBC) [34]. We used a previously described established assay for CH50 determination [35–37]. In short, human serum is titrated to obtain the fraction of serum which causes lysis of 50% of the RBCs as measured by the hemoglobin released into the supernatant. Antibody-sensitized sheep erythrocytes (Complement Tech, Tyler TX) were centrifuged for 3 min at 1000 g. The supernatant was removed and replaced with an equal volume of fresh gelatin veronal buffered saline containing 0.15 mM calcium chloride and 0.5 mM magnesium chloride (GVB++; Complement Tech, Tyler Tx). The sheep erythrocytes were at a concentration of 5 x 10^8^ cells per ml. Human sera samples were diluted in 10 serial dilutions (2-fold each) in GVB++. Sheep erythrocytes (100 μl), 50 μl of GVB++ and 50 μl of the serum sample were added into 96-well tissue culture treated plates (USA Scientific) and mixed by pipetting up and down three times. The plate was incubated at 37°C for 1 h. After incubation the plate was centrifuged at 1000 g for 3 min and 100 μl of the supernatant was transferred to a new plate without disturbing the pellet. The absorbance of the hemoglobin released into the supernatant was read at 412 nm with the Infinite® 200 PRO (Tecan Life Sciences, Switzerland). Assay controls consisted of GVB++ buffer only as the background of the plate, sheep erythrocytes with GVB++ as the background of lysis, and sheep erythrocytes lysed with Triton X100 for total lysis. Additionally, we included the same sample (standard) for inter-assay standardization. Only assay results with a range of values +/- 20% within the standard sample were included in the final analysis.

### Statistics

Statistics were calculated with Prism Software (GraphPad; version 8.0). Descriptive statistics were used to evaluate the mean of age between the AChR MG and HC cohort and to set the CH50 assay cut-off for low complement activity (mean +/- 2 SD). Spearman correlation was used to assess the correlation of AChR autoantibody titers and disease burden (measured by MGC score) to CH50 assay values. Bonferroni correction was used to adjust for multiple tests.

## Results

**Fig 1:**
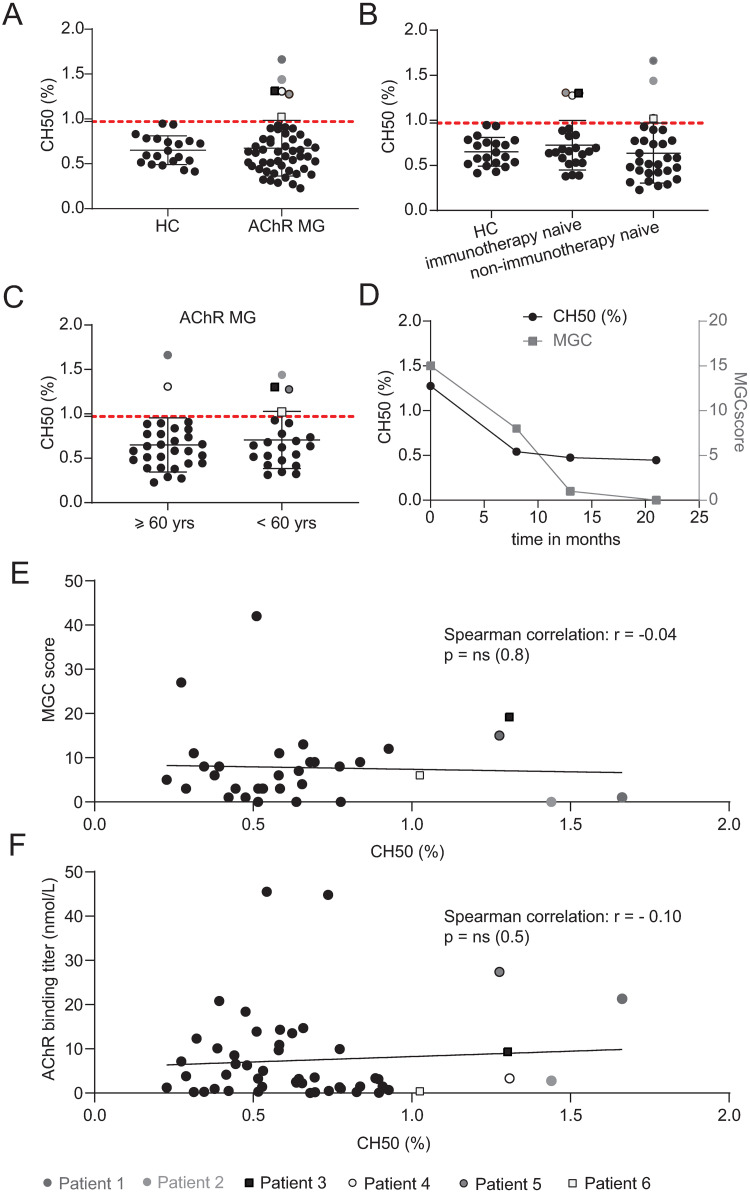
Complement activity in AChR MG does not associate with clinical status or autoantibody titer. Complement activity in the serum of AChR MG patients was measured by CH50 hemolysis assay. The assay measures the activation of the classical complement pathway by testing the ability of the complement components of sera samples to lyse antibody-sensitized sheep erythrocytes. The CH50 values are given as the percentage (%) of serum needed to lyse 50% of sheep erythrocytes. (A) AChR MG patients (N = 51) and healthy controls (N = 20) were measured by CH50 hemolysis assay. (B) Comparison of the complement activity between immunotherapy naïve (N = 22) vs non-immunotherapy naïve (N = 29) AChR MG patients. (C) Comparison of the complement activity between AChR MG patients 60 years of age and older (N = 29) vs AChR MG patients younger than 60 years of age (N = 22). (D) Serial samples of patient 5 (Table 3) were measured by CH50 hemolysis assay and compared to the corresponding disease burden (MGC score) at the time of each collection. The X axis shows the time in months since the first sample collection, the left Y axis shows the CH50 values (%) and the right Y axis shows the Myasthenia Gravis Composite (MGC) score. (E) Correlation of complement activity with the MGC score. The MGC score values were available for 33 of the 51 patients. (F) Correlation of complement activity with the AChR antibody titer (N = 51). The linear regression is shown with Spearman correlation values. Patients 1–6 (Table 2) are individually illustrated (see legend) with their corresponding MGC score (E) or antibody titer (F) if values were available. Values higher than the mean + 2SD of the HC controls (indicated by the horizontal dotted line) were considered reduced (A-C).

AChR MG patients (N = 51, mean age: 59.3 +/- 18.8 yrs; (Table 1)) and healthy controls (HC;N = 20, mean age: 34.4 +/- 13.3 yrs) were tested for complement activity by hemolytic CH50 assay. The majority of AChR MG samples (88.2%) had complement activity within the range defined by the HCs (Fig 1A). Six AChR MG samples (11.8%) had reduced complement activity (Fig 1A; Table 2). Three out of the six samples with reduced complement activity were immunotherapy naïve at the time of collection (Table 2).

**Table 2:** Characteristics of six AChR MG patients with reduced complement activity.

Subject ID	Age at TOC, Sex	EOMG/LOMG	Antibody titer	MGFA class at TOC	Treatment at TOC	Immunotherapy naïve at TOC	Thymectomy
Patient 1	72, M	LOMG	21.3	I	Mestinon 180 mg/d	No	-
Patient 2	36, F	EOMG	2.79	0	-	No	4
Patient 3	46, F	EOMG	9.21	IIIA	-	Yes	1.5
Patient 4	72, M	LOMG	3.31	I	-	Yes	-
Patient 5	32, M	EOMG	27.4	IIIA	-	Yes	-
Patient 6	57, M	LOMG	0.43	I	Mestinon 540 mg/d	No	-

Antibody titer was measured at the Mayo Clinic Laboratory; the unit is nmol/L, the cut off for negativity is ≤ 0.02 nmol/L. The values for thymectomy represent the time in years since thymectomy. MGFA class = Myasthenia Gravis Foundation of America classification; TOC = time of collection; EOMG = early-onset myasthenia gravis; LOMG = late-onset MG.

**Fig 2:**
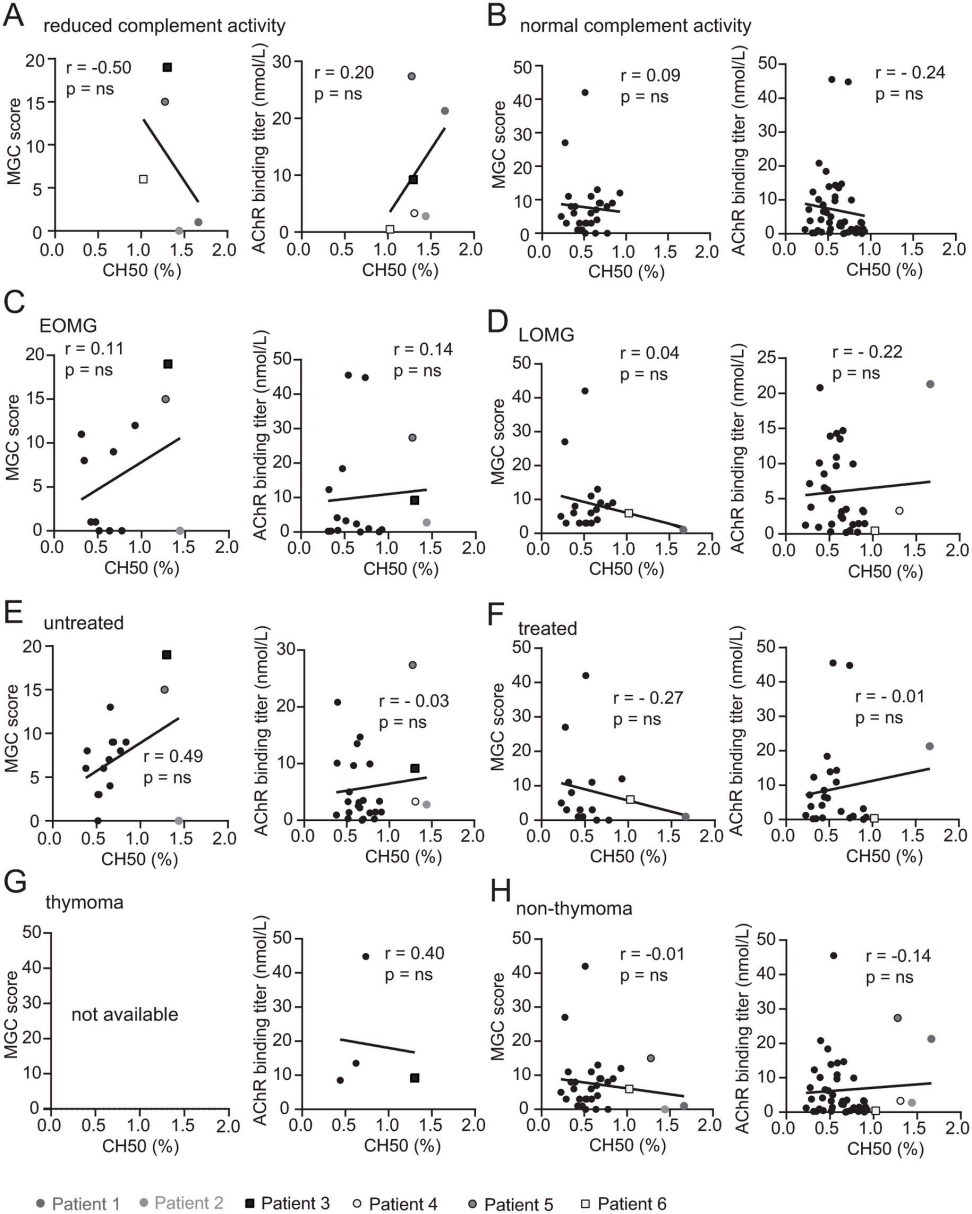
Correlation between complement activity and MG patient subcohort demographics. Correlation tests between complement activity to clinical status and autoantibody titer in different MG patient subcohorts defined by complement activity, age of disease onset, treatment status, and thymoma. (A-H) Correlation of complement activity with MGC score (first and third columns) and AChR antibody titer (second and fourth columns). The subcohorts are defined by complement activity (A and B), age of disease onset (early onset MG (EOMG; C); late onset MG (LOMG; D)), treatment status (E and F) and thymoma status (G and H). Limited specimens (n = 1) in the thymoma category with matching MGC scores prohibited correlative analysis (G left panel). The linear regression is shown with Spearman correlation values. The Bonferroni correction was used to adjust for multiple tests. Patient 1–6 (Table 2) are individually illustrated (see legend) for each panel.

The largest subcohort within our samples consisted of immunotherapy naïve samples (43.1%). We first compared the complement activity between immunotherapy naïve and non-immunotherapy naïve samples and found no significant difference between these two groups (Fig 1B). Recent studies indicate that complement activity increases with age; one such study found differences when comparing the values of participants over 60 years of age to younger participants [38]. This age-dependent increased complement activity could possibly conceal reduced levels. Our patient cohort included patients (57%) at the age of 60 years and older. However, we found no significant difference of complement activation between MG patients 60 years of age and older at time of collection to MG patients younger than 60 years of age **(**Fig 1C). Serial samples of one patient with reduced complement activity were tested to investigate how the activity changed over time. The complement activity and disease burden (MGC score) of this patient normalized over the time course of 21 months (Fig 1D; Table 3). Next, we investigated whether complement activity within our cohort of 51 patients associates with MG disease burden or the circulating levels of AChR autoantibodies. No association (ns; p = 0.8) between disease burden (MGC score) and complement activity was observed (Fig 1E). Similarly, no significant (ns; p = 0.5) correlation between complement activity and AChR autoantibody titer was observed (Fig 1F). We further analyzed the correlation of complement activity to MGC score and AChR autoantibody titer by comparing subcohorts defined by complement activity, age of disease onset (early onset MG (EOMG) for onset before the age of 50 years; late onset MG (LOMG) for onset on or after the age of 50 years), treatment status and thymoma and found no significant correlations (Fig 2A–2H).

**Table 3:** Patient characteristics during longitudinal sample collection.

Sample Collection (months)	Antibody titer	MGFA class, and MGC score at TOC	Treatment at TOC
0	27.4	IIIA; 15	-
8	45.5	IIB; 8	Pred 20 mg/d
13	18.4	I; 1	Pred 10 mg/d, IVIg
21	10.3	0; 0	IVIg

The time point 0 is normalized to indicate the first sample (Patient 5 (Table 1)) in the series. The values for timepoints within the serial sample represent the time in months since the first sample. Antibody titer was measured at the Mayo Clinic Laboratory; the unit is nmol/L, the cut off for negativity is ≤ 0.02 nmol/L. TOC = time of collection; MGFA class = Myasthenia Gravis Foundation of America classification; MGC score = MG Composite score; Pred = prednisone.

## Discussion

In this study we measured the total complement activity by the CH50 hemolysis assay. The findings suggest that complement activity in most AChR MG patients is within the range defined by HC. Decreased complement activity was observed in a small subgroup. While secondary deficiencies of complement activity can arise during episodes of autoimmune disease activity, we observed no association between complement activity and AChR autoantibody titer or between complement activity and disease activity.

We chose to focus on the CH50 hemolysis assay because it is a functional assay that provides a quantitative measurement of the classical pathway overall activity. While the CH50 hemolysis assay is widely used and highly reliable, there are limitations that must be recognized. One limitation of the CH50 hemolysis assay is that it is susceptible to acute inflammatory processes which lead to increased complement activity possibly concealing reduced activity [39]. The patients in our study cohort did not have any apparent acute non-MG related inflammation. Another limitation is that while the assay measures the functionality of the classical complement pathway, it cannot be used to identify altered levels of specific proteins within that pathway. Circulating levels of C3 and C4 have been investigated in MG with divergent results. Their levels were found to be the same as those found in HC [40, 41] or conversely, the levels of C3 and C4 were reduced in MG patients [42, 43]. Additionally, increased levels of soluble C5b-9 were found in MG patients in comparison to HCs [41] and C5a levels showed a positive correlation with disease severity [40]. While we identified six patients with diminished complement activity, determining which components were reduced was outside of the scope of the study.

Eculizumab, a therapeutic complement inhibitor, was demonstrated to be effective in a phase 3 trial with subsequent approval for its use in treating AChR receptor antibody-positive MG [44, 45]. Clinical response to this treatment can be heterogeneous; many patients respond well or show a delayed response while, others respond poorly. The mechanisms underlying the variable response are not known. However, there is a need for further understanding so that responses can be better predicted. The assays that are routinely used to diagnose MG, measure the capability of the polyclonal serum derived autoantibodies to bind to the AChR, but not their pathogenic properties. Three major pathogenic mechanisms have been identified for AChR autoantibodies. The first describes AChR autoantibodies blocking the access of acetylcholine to the AChR and thus hindering the neuromuscular signal transduction [46]. The second, termed antigen modulation, describes AChR receptor crosslinking followed by receptor internalization resulting in a reduced number of available receptors [47]. The third involves activation of the classical complement pathway by AChR autoantibodies [22]. There may also be AChR autoantibodies that bind the receptor in the clinical diagnostic assay but have no pathogenic capacity. We propose that the discordance between titer and disease severity and between titer and treatment response may be due to the limitations of diagnostic assays. Specifically, because they measure only binding, they are wholly unable to discriminate between detection of AChR autoantibodies and their pathogenic mechanism or if they are nonpathogenic. Currently, there is no established approach for measuring the complement activating properties of AChR autoantibodies. Given that complement activity in most MG patients is normal and lacks an association with disease activity, such a tool would provide valuable biomarker data for identifying patients expected to respond to complement inhibitor-based treatments.

## Supporting information

S1 Dataset (PDF)
