# Supplementary material for: Myasthenia gravis complement activity is independent of autoantibody titer and disease severity
Source: PLoS One. 2022 Mar 15;17(3):e0264489. doi: 10.1371/journal.pone.0264489 (PMC8923450; doi:10.1371/journal.pone.0264489)
Supplement: S1 Dataset — (PDF) [file pone.0264489.s001.pdf]

Sample ID	EOMG or LOMG	Thymectomy (Yes/No)	Hyperplasia (Yes/No)	Thymoma (Yes/No)	MGC score at TOC	Immunotherapy Naive (Yes/No)	Antibody titer (nmol/L)	CH50 (%)
**MG1**	LOMG	No	-	-	-	No	0.47	0.74
**MG2***	EOMG	Yes	Yes	No	-	No	4.15	0.42
**MG3**	LOMG	No	-	-	-	Yes	2.54	0.64
**MG4**	LOMG	No	-	-	-	No	3.15	0.89
**MG5**	EOMG	Yes	-	-	-	No	12.3	0.32
**MG6**	LOMG	Yes	No	Yes	-	Yes	13.5	0.62
**MG7**	LOMG	No	-	-	-	Yes	0.24	0.83
**MG8**	LOMG	No	-	-	-	Yes	10.1	0.39
**MG9**	EOMG	Yes	No	Yes	-	No	44.8	0.74
**MG10**	EOMG	No	-	-	-	No	0.05	0.90
**MG11**	LOMG	Yes	No	Yes	-	No	8.53	0.44
**MG12**	LOMG	No	-	-	-	Yes	3.35	0.89
**MG13^**	EOMG	Yes	No	No	15	Yes	27.4	1.28
**MG14**	LOMG	No	-	-	-	Yes	0.2	0.70
**MG15**	LOMG	No	-	-	7	Yes	3.17	0.64
**MG16#**	EOMG	Yes	No	No	11	No	0.22	0.31
**MG17**	LOMG	No	-	-	3	Yes	0.3	0.52
**MG18**	LOMG	No	-	-	3	No	14.3	0.58
**MG19**	LOMG	No	-	-	1	No	21.3	1.66
**MG20#**	EOMG	Yes	No	No	8	No	0.25	0.35
**MG21**	LOMG	No	-	-	9	Yes	3.5	0.69
**MG22**	LOMG	No	-	-	9	Yes	1.47	0.84
**MG23***	EOMG	Yes	Yes	No	0	No	3.28	0.52
**MG24**	EOMG	No	-	-	9	Yes	0	0.68
**MG25**	LOMG	No	-	-	11	No	10.9	0.58
**MG26%**	EOMG	Yes	Yes	No	12	No	0.68	0.93
**MG27@**	LOMG	No	-	-	13	Yes	14.7	0.66
**MG28!**	LOMG	No	-	-	27	No	7.16	0.27
**MG29**	LOMG	No	-	-	3	No	6.58	0.44
**MG30%**	EOMG	Yes	Yes	No	1	No	0.49	0.42
**MG31@**	LOMG	No	-	-	3	No	3.81	0.29
**MG32!**	LOMG	No	-	-	5	No	1.23	0.23
**MG33**	LOMG	No	-	-	6	Yes	0.95	0.38
**MG34**	LOMG	No	-	-	6	Yes	9.67	0.58
**MG35**	LOMG	No	-	-	8	Yes	20.8	0.39
**MG36**	LOMG	No	-	-	6	No	0.43	1.03
**MG37“**	LOMG	No	-	-	-	No	1.32	0.77
**MG38***	EOMG	Yes	Yes	No	0	No	2.79	1.44
**MG39**	LOMG	No	-	-	4	Yes	2.22	0.65
**MG40**	LOMG	No	-	-	3	Yes	1.38	0.53
**MG41**	LOMG	No	-	-	-	No	6.26	0.48
**MG42“**	LOMG	No	-	-	8	No	9.94	0.77
**MG43**	LOMG	No	-	-	3	Yes	5.02	0.53
**MG44**	LOMG	No	-	-	-	Yes	3.31	1.31
**MG45**	LOMG	No	-	-	42	No	13.9	0.51
**MG46**	EOMG	Yes	No	Yes	19	Yes	9.21	1.30
**MG47**	LOMG	No	-	-	-	Yes	1.47	0.91
**MG48^**	EOMG	Yes	No	No	1	No	18.4	0.48
**MG49^**	EOMG	Yes	No	No	-	No	45.5	0.54
**MG50***	EOMG	Yes	Yes	No	0	No	2.37	0.64
**MG51***	EOMG	Yes	Yes	No	0	No	0.97	0.78
**HC1**	-	-	-	-	-	-	-	0.58
**HC2**	-	-	-	-	-	-	-	0.48
**HC3**	-	-	-	-	-	-	-	0.43

**HC4**	-	-	-	-	-	-	-	0.41
**HC5**	-	-	-	-	-	-	-	0.95
**HC6**	-	-	-	-	-	-	-	0.52
**HC7**	-	-	-	-	-	-	-	0.59
**HC8**	-	-	-	-	-	-	-	0.94
**HC9**	-	-	-	-	-	-	-	0.73
**HC10**	-	-	-	-	-	-	-	0.78
**HC11**	-	-	-	-	-	-	-	0.83
**HC12**	-	-	-	-	-	-	-	0.59
**HC13**	-	-	-	-	-	-	-	0.78
**HC14**	-	-	-	-	-	-	-	0.65
**HC15**	-	-	-	-	-	-	-	0.75
**HC16**	-	-	-	-	-	-	-	0.72
**HC17**	-	-	-	-	-	-	-	0.49
**HC18**	-	-	-	-	-	-	-	0.74
**HC19**	-	-	-	-	-	-	-	0.54
**HC20**	-	-	-	-	-	-	-	0.53

Table of Minimal Data Set. Serial samples are indicated with the same symbol (!, @, #, %, ^, *, “)
